# Predictors of Short‐ and Long‐Term Major Adverse Cardiac and Cerebrovascular Events (MACCE) After Percutaneous Coronary Intervention: A Cohort Study on the Data of National Persian Registry of Cardiovascular Disease (N‐PROVE)

**DOI:** 10.1002/hsr2.73272

**Published:** 2026-09-16

**Authors:** Nahid Azdaki, Seyed Ali Moezi Bady, Toba Kazemi, Marzieh Azimizadeh, Hamidreza Mohammadi, Alireza Khosravi Farsani, Nizal Sarrafzadegan, Maryam Boshtam, Pedro Manuel Marques‐Vidal

**Affiliations:** ^1^ Cardiovascular Diseases Research Center Birjand University of Medical Sciences Birjand Iran; ^2^ Clinical Research Development Unit, Razi Hospital Birjand University of Medical Sciences Birjand Iran; ^3^ Yazd Cardiovascular Research Center, Non‐communicable Diseases Research Institute Shahid Sadoughi University of Medical Sciences Yazd Iran; ^4^ Hypertension Research Center, Cardiovascular Research Institute Isfahan University of Medical Sciences Isfahan Iran; ^5^ Iranian Network of Cardiovascular Research Tehran Iran; ^6^ Isfahan Cardiovascular Research Center, Cardiovascular Research Institute Isfahan University of Medical Sciences Isfahan Iran; ^7^ Department of Medicine, Internal Medicine Lausanne University Hospital and University of Lausanne Office BH10‐642, 46 Rue du Bugnon Lausanne 1011 Switzerland

**Keywords:** coronary artery disease, diabetes mellitus, major adverse cardiac and cerebrovascular events, myocardial infarction, percutaneous coronary intervention

## Abstract

**Background and Aims:**

Major adverse cardiac and cerebrovascular events (MACCE) are significant complications following percutaneous coronary intervention (PCI). Identifying the predictors of these events can aid in improving outcomes, especially in specific populations such as the Iranian cohort. This study aims to investigate the incidence and predictors of short‐ and long‐term MACCE after PCI in patients from the National Persian Registry of Cardiovascular Disease (N‐PROVE).

**Methods:**

We conducted a cohort study involving 1921 patients who underwent PCI for acute coronary syndrome at Razi Hospital, Birjand, Iran. Data on demographics, medical history, procedural details, and follow‐up outcomes were collected. The primary endpoint was the occurrence of MACCE, including all‐cause mortality, myocardial infarction (MI), cerebrovascular accident (CVA), and target‐vessel revascularization (TVR), at 1‐, 6‐, and 12‐months post‐PCI. Predictors of MACCE were identified using univariate and multivariate Cox proportional hazards models.

**Results:**

MACCE occurred in 8.5% of patients at 1 month, 13.7% at 6 months, and 17.9% at 12 months post‐PCI. Independent predictors of MACCE included age ≥ 75 years, female sex, diabetes mellitus (DM), multi‐vessel disease (MVD), and poor TIMI flow. Multivariate analysis confirmed DM (HR 1.52, 95% CI 1.07–2.23), 2VD (HR 1.59, 95% CI 1.05–2.42), 3VD (HR 1.55, 95% CI 1.01–2.75), and TIMI flow 0‐I (HR 2.31, 95% CI 1.53–3.49) as significant predictors.

**Conclusions:**

The study highlights key predictors of MACCE, particularly age, sex, DM, and TIMI flow. These findings underscore the need for tailored interventions for high‐risk groups, especially elderly patients and those with MVD, to improve long‐term outcomes following PCI.

## 1 Introduction

Coronary artery disease (CAD) is the primary cause of death globally, contributing to over 37.4% of fatalities in the United States and nearly half of all deaths in Iran due to cardiovascular conditions [1]. Percutaneous coronary intervention (PCI) is a widely utilized treatment for CAD, which has significantly enhanced survival rates and reduced disability and mortality associated with CAD [2]. Despite its frequent use as the predominant method of myocardial revascularization, the risk of major adverse cardiac and cerebrovascular events (MACCE) remains a critical concern that can influence patient outcomes. MACCE typically encompasses a range of conditions, including heart failure, myocardial infarction (MI), cerebrovascular accident (CVA), stable angina, cardiovascular rehospitalization, repeat PCI, repeat CABG, and mortality, all of which have been extensively studied across different contexts [3].

As the demand for PCI continues to grow, the development of models to predict the occurrence of MACCE has become a clinical challenge, essential for aiding early intervention, and guiding treatment and rehabilitation plans for both physicians and patients. Various studies have explored the risk factors associated with MACCE, identifying factors such as demographic characteristics and medical histories, including female sex, advanced age, obesity, dyslipidemia, smoking, hypertension (HTN), and diabetes mellitus (DM) [4].

Various studies have investigated the predictor variables of clinical outcomes following PCI. However, these PCI predictive models have primarily focused on short‐term or in‐hospital outcomes and have often examined long‐term clinical outcomes separately [5, 6, 7]. There has been less emphasis on simultaneous assessments of both short‐term and long‐term outcomes. Therefore, we aim to evaluate the predictors of MACCE after PCI at 1, 6, and 12‐month intervals within a large‐scale Iranian population participating in the Persian Cardiovascular Disease Registry.

## 2 Patients and Methods

### 2.1 Study Population

This cohort study was conducted using data from the National Persian Registry of Cardiovascular Disease (N‐PROVE), a population‐based registry for evaluating efficacy and outcomes of various CVD interventions [8]. after a successful pilot study known as the Persian Registry Of CardioVascular DiseasE (PROVE), conducted since 2014 [9]. A national registry of N‐PROVE was implemented in 19 hospitals located in 7 provinces in Iran. Patients undergoing coronary angiography or PCI were included in the registry. Five questionnaires, including baseline patient characteristics, procedure details, discharge status, and follow‐up at 1, 6, and 12 months after the intervention, were completed.

### 2.2 Ethical Approval

The present study was approved by the Birjand University of Medical Sciences (ethics code: IR.BUMS.REC.1401.331) and conducted based on the Declaration of Helsinki on medical research. Informed consent was obtained from eligible individuals. The data collection process ensured complete anonymity, with no personally identifying information included in the individual data.

### 2.3 Inclusion and Exclusion Criteria

Patients who underwent PCI for acute coronary syndrome (ACS) at Razi Hospital, affiliated with Birjand University of Medical Sciences in East Iran, from March 2017 to October 2019 were eligible.

The exclusion criteria encompassed refusal to consent to invasive treatment, active or recent internal hemorrhage, a history of bleeding subsequent to the administration of non‐steroidal anti‐inflammatory drugs (NSAIDs), known coagulopathies, the existence of an intracerebral mass or aneurysm, intolerance or hypersensitivity to aspirin or clopidogrel, cardiogenic shock upon admission, non‐cardiac conditions that might hinder compliance with the study protocol, and any concurrent condition linked to a diminished short‐term life expectancy.

### 2.4 Data Collection

The diagnosis of patients was validated using clinical examinations, medical history, laboratory tests, and electrocardiograms (ECG). The patient data required for this study were retrieved from the N‐PROVE registry database. Data including demographic features (age, sex, and Body Mass Index (BMI)), smoking, use of opium, family history of CAD, and medical history including CVA, DM, dyslipidemia, CABG, and PCI, were collected when the patient was in the hospital. However, the follow‐up forms for post‐PCI cardiovascular events are collected via phone interviews. If a patient reports a positive event, they are requested to provide supporting documents and medical records for verification. The events' ascertainment committee reviews the relevant documents and determines the occurrence of CVD events by consensus. BMI was defined as weight in kilograms divided by the square of height in meters. Patients were considered smokers if they were current smokers.

Data related to interventional therapy, including number of vessels involved, culprit vessels, and pre‐ and postprocedural thrombolysis in myocardial infarction (TIMI) flow grade, were obtained.

### 2.5 Endpoints and Definitions

The primary endpoint of this study was the evaluation of MACCE at 1, 6, and 12‐month intervals following PCI. MACCE was defined as all‐cause mortality, MI, CVA, and target‐vessel revascularization (TVR). MI was defined as the presence of clinical symptoms, ECG changes, and elevation of CK‐MB, troponin I or T levels > 3 fold of the upper limit of the normal value. TVR was defined as the repeated revascularization of the target vessel via PCI or CABG. CVA was defined as a history of symptomatic brain dysfunction from a vascular cause. The degree of stenosis was classified as significant if the patient had > 70% diameter stenosis according to the American Heart Association classification. Flow grades were assessed according to TIMI criteria [10].

### 2.6 Interventional Procedure and Medical Treatment

All patients received aspirin (300 mg) orally, a clopidogrel loading dose (300 mg), and weight‐adjusted unfractionated heparin (80–100 U/kg) intravenously prior to the surgery. PCI was conducted utilizing conventional methodologies. The postprocedural antiplatelet protocol included lifetime aspirin and clopidogrel (75 mg/day) for a minimum duration of 1 year. The medicinal therapy included statins, antiplatelet agents, β‐blockers, nitrates, and renin‐angiotensin system inhibitors.

### 2.7 Statistical Analysis

All statistical analyses were conducted using SPSS software version 23.0 (IBM Corp., Armonk, NY, USA). Continuous variables were presented as mean ± standard deviation (SD), and an independent *t*‐test was used to compare means between the groups with and without events. Categorical variables were expressed as frequencies and percentages, and *χ* ^2^ or Fisher's exact tests were used to compare the two groups. Missing data were handled using a complete‐case approach; of 2073 patients, 152 (7.3%) with incomplete baseline data or undocumented follow‐up were excluded, leaving 1921 patients for analysis. The Cox proportional hazards regression model was used to determine predictors of MACCE occurrence at 1‐, 6‐, and 12‐months after PCI. First, factors associated with MACCE were evaluated by univariate analysis. Second, all possible risk factors with a univariate *p* value < 0.1 were used in the multivariate Cox regression model. Hazard ratios (HR), accompanied by 95% confidence intervals (CI) and *p* value, were presented. A two‐tailed *p* value below 0.05 was deemed statistically significant. Kaplan–Meier curves were generated from the date of the PCI procedure to the occurrence of MACCE and were analyzed between groups using the log‐rank test.

## 3 Results

The analysis included 1921 patients, 1358 males (70.7%; mean age 62.35 ± 11.57 years) and 563 females (29.3%; mean age 65.1 ± 10.45 years), who underwent PCI. Baseline demographic and clinical characteristics of the patients who developed/did not develop MACCE are summarized in Table 1. MACCE occurred in 163 (8.5%) patients during the first month, 263 (13.7%) patients during the 6‐month follow‐up, and 344 (17.9%) patients during the 12‐month follow‐up. Patients with MACCE were generally older and more likely to be female at all follow‐up intervals. Regarding medical history, DM was more common among MACCE patients, particularly at 6 and 12 months (both *p*= 0.01). At 12‐month follow‐up, HTN was also more prevalent among MACCE patients (55.2% vs. 48.4%, *p*= 0.02). Coronary angiography revealed a higher incidence of multi‐vessel disease (MVD) and TIMI flow < 3 both pre‐ and post‐procedure in MACCE patients across all follow‐up periods. No significant differences were observed in the distribution of culprit vessels between patients with and without MACCE (Table 1).

**Table 1:** Baseline characteristics.

	1‐Month			6‐Months			12‐Months		
	MACCE (*n* = 163)	Without MACCE (*n* = 1758)	*p* value	MACCE (*n* = 263)	Without MACCE (*n* = 1658)	*p* value	MACCE (*n* = 344)	Without MACCE (*n* = 1577)	*p* value
Age (years)	64.31 ± 11.98	62.17 ± 11.51	0.001	65.15 ± 11.81	61.9 ± 11.47	< 0.001	64.97 ± 11.42	61.78 ± 11.52	< 0.001
Sex, female	56 (34.4)	507 (28.8)	0.1	94 (35.7)	469 (28.3)	0.01	122 (35.5)	441 (28.0)	0.01
BMI, kg/m^2^	25.16 ± 4.5	25.43 ± 4.2	0.02	25.19 ± 4.52	25.11 ± 4.21	0.8	25.18 ± 4.53	25.19 ± 4.2	0.9
Medical history									
Prior CVA	5 (3.1)	31 (1.8)	0.2	7 (2.7)	29 (1.8)	0.3	8 (2.3)	28 (1.8)	0.5
DM, *n* (%)	49 (30.2)	432 (24.7)	0.1	82 (31.3)	399 (24.2)	0.01	106 (31.1)	375 (23.9)	0.01
Dyslipidemia	32 (19.9)	383 (22.4)	0.4	52 (20.5)	363 (22.5)	0.5	72 (21.8)	343 (22.3)	0.8
HTN	82 (50.6)	865 (49.5)	0.8	141 (54.2)	806 (48.9)	0.1	187 (55.2)	760 (48.4)	0.02
Family history of CAD	20 (12.3)	300 (17.2)	0.1	35 (13.5)	285 (17.3)	0.1	46 (13.6)	274 (17.5)	0.08
Prior CABG	5 (3.1)	71 (4.1)	0.5	12 (4.6)	64 (3.9)	0.6	17 (5.0)	59 (3.8)	0.3
Prior PCI	23 (14.1)	260 (14.9)	0.8	48 (18.3)	235 (14.2)	0.08	62 (18.2)	221 (14.1)	0.05
Opium	48 (29.4)	461 (26.3)	0.4	71 (27.0)	438 (26.5)	0.9	92 (26.8)	417 (26.5)	0.9
Smoking	32 (19.6)	277 (15.9)	0.2	46 (17.5)	263 (16.0)	0.5	59 (17.2)	250 (16.0)	0.6
Number of vessels involved									
SVD	44 (27.0)	677 (38.5)	0.01	67 (25.5)	654 (39.4)	< 0.001	98 (28.5)	623 (39.5)	< 0.001
2VD	65 (39.9)	626 (35.6)		111 (42.2)	580 (35.0)		134 (39.0)	557 (35.3)	
3VD	54 (33.1)	455 (25.9)		85 (32.3)	424 (25.6)		112 (32.6)	397 (25.2)	
Culprit vessel^a^									
LM	0	5 (0.3)	0.85	1 (0.4)	4 (0.2)	0.9	1 (0.3)	4 (0.3)	0.8
LAD	69 (45.4)	728 (42.8)		111 (45.1)	686 (42.7)		143 (43.9)	654 (42.8)	
RCA	47 (30.9)	533 (31.3)		77 (31.3)	503 (31.3)		98 (30.1)	482 (31.6)	
LCX	32 (21.1)	363 (21.3)		50 (20.3)	345 (21.5)		74 (22.7)	321 (21.0)	
Ramus	2 (1.3)	50 (2.9)		5 (2.0)	47 (2.9)		8 (2.5)	44 (2.9)	
Diagonal	2 (1.3)	22 (1.3)		2 (0.8)	22 (1.4)		2 (0.6)	22 (1.4)	
TIMI (preprocedural) flow^a^									
0‐І	76 (50.3)	601 (35.5)	< 0.001	116 (47.9)	561 (35.0)	< 0.001	138 (43.0)	539 (35.4)	0.001
II	37 (24.5)	370 (21.9)		55 (22.7)	352 (22.0)		77 (24.0)	330 (21.7)	
III	38 (25.2)	722 (42.6)		71 (29.3)	689 (43.0)		106 (33.0)	654 (42.9)	
TIMI (postprocedural) flow^b^									
0‐I	11 (7.2)	29 (1.7)	< 0.001	17 (6.9)	23 (1.4)	< 0.001	18 (5.5)	22 (1.4)	< 0.001
II	14 (9.2)	54 (3.2)		17 (6.9)	51 (3.2)		20 (6.1)	48 (3.1)	
III	127 (83.6)	1618 (95.1)		1533 (86.2)	1533 (95.4)		288 (88.3)	1457 (95.4)	

*Note:* Data presented as mean ± standard deviation or number (%).

Abbreviations: 2VD, two vessel disease; 3VD, three vessel disease; BMI, body mass index; CABG, coronary artery bypass grafting; CAD, coronary artery disease; CVA, cerebral vascular accident; DM, diabetes mellitus; HTN, hypertension; LCX, left circumflex artery; LM, left main; PCI, percutaneous coronary intervention; RCA, right coronary artery; SVD, single vessel disease; TIMI, thrombolysis in myocardial infarction.

^a^Data available for culprit vessel, *n *= 1853; TIMI (preprocedural) flow, *n *= 1844; TIMI (preprocedural) flow, *n * = 1853.

In the univariate Cox regression analysis, age ≥ 75 (HR 1.47, 95% CI 1.02–2.13), 2VD (HR 1.56, 95% CI 1.07–2.3), 3VD (HR 1.8, 95% CI 1.2–2.7), TIMI flow grade 0‐І (HR 2.32, 95% CI 1.6–3.4), and TIMI flow grade П (HR 1.85, 95% CI 1.18–2.9) were independently risk factors for 1‐months MACCE occurrence after PCI. Similar observations were recorded in the medium and longer term at 6 and 12‐months, respectively. Additionally, female sex, and DM were risk factors for MACCE at 6 and 12‐months (Table 2).

**Table 2:** Univariable analyses of associations between variables and the occurrence of MACCE at 1, 6, and 12‐months after PCI.

	1‐Month		6‐Months		12‐Months	
	HR (CI 95%)	*p* value	HR (CI 95%)	*p* value	HR (CI 95%)	*p* value
Age (Ref, <75)						
≥75	1.47 (1.02–2.13)	0.04	1.66 (1.25–2.20)	0.001	1.48 (1.15–1.92)	<0.001
Female	1.26 (0.91–1.74)	0.15	1.36 (1.05–1.75)	0.02	1.35 (1.08–1.69)	0.01
BMI	1.01 (0.98–1.05)	0.4	0.99 (0.90–1.03)	0.8	1.01 (0.97–1.02)	0.9
CVA	1.74 (0.71–4.25)	0.2	1.51 (0.71–3.20)	0.3	1.32 (0.65–2.67)	0.4
DM	1.30 (0.93–1.80)	0.1	1.38 (1.06–1.80)	0.01	1.38 (1.10–1.70)	0.005
Dyslipidemia	0.86 (0.58–1.26)	0.4	0.89 (0.65–1.20)	0.4	0.96 (0.74–1.25)	0.9
HTN	1.04 (0.76–1.41)	0.8	1.21 (0.94–1.54)	0.13	1.27 (1.02–1.57)	0.03
Family history of CAD	0.68 (0.43–1.09)	0.1	0.75 (0.53–1.07)	0.12	0.76 (0.56–1.04)	0.08
Prior CABG	0.76 (0.31–1.86)	0.55	1.16 (0.65–2.07)	0.6	1.30 (0.78–2.08)	0.3
Prior PCI	0.94 (0.60–1.47)	0.8	1.31 (0.96–1.79)	0.09	1.30 (0.99–1.73)	0.05
Opium	1.16 (0.83–1.62)	0.4	1.03 (0.78–1.35)	0.8	1.02 (0.80–1.30)	0.9
Smoking	1.29 (0.90 – 1.90)	0.2	1.12 (0.81–1.54)	0.5	1.10 (0.83–1.45)	0.5
Number of vessels involved (ref, SVD)						
2VD	1.56 (1.07–2.30)	0.02	1.78 (1.32–2.42)	0.001	1.50 (1.15–1.93)	0.003
3VD	1.80 (1.20–2.70)	0.001	1.89 (1.37–2.60)	0.001	1.70 (1.30–2.26)	0.001
Preprocedural TIMI flow (ref, III)						
0–I	2.32 (1.60–3.40)	0.001	1.93 (1.44–2.60)	0.001	1.55 (1.20–2.00)	0.001
II	1.85 (1.18–2.90)	0.01	1.49 (1.05–2.12)	0.02	1.41 (1.05–1.90)	0.02

*Note:* The results are expressed as hazard ratios and 95% confidence intervals.

**Figure 1:**
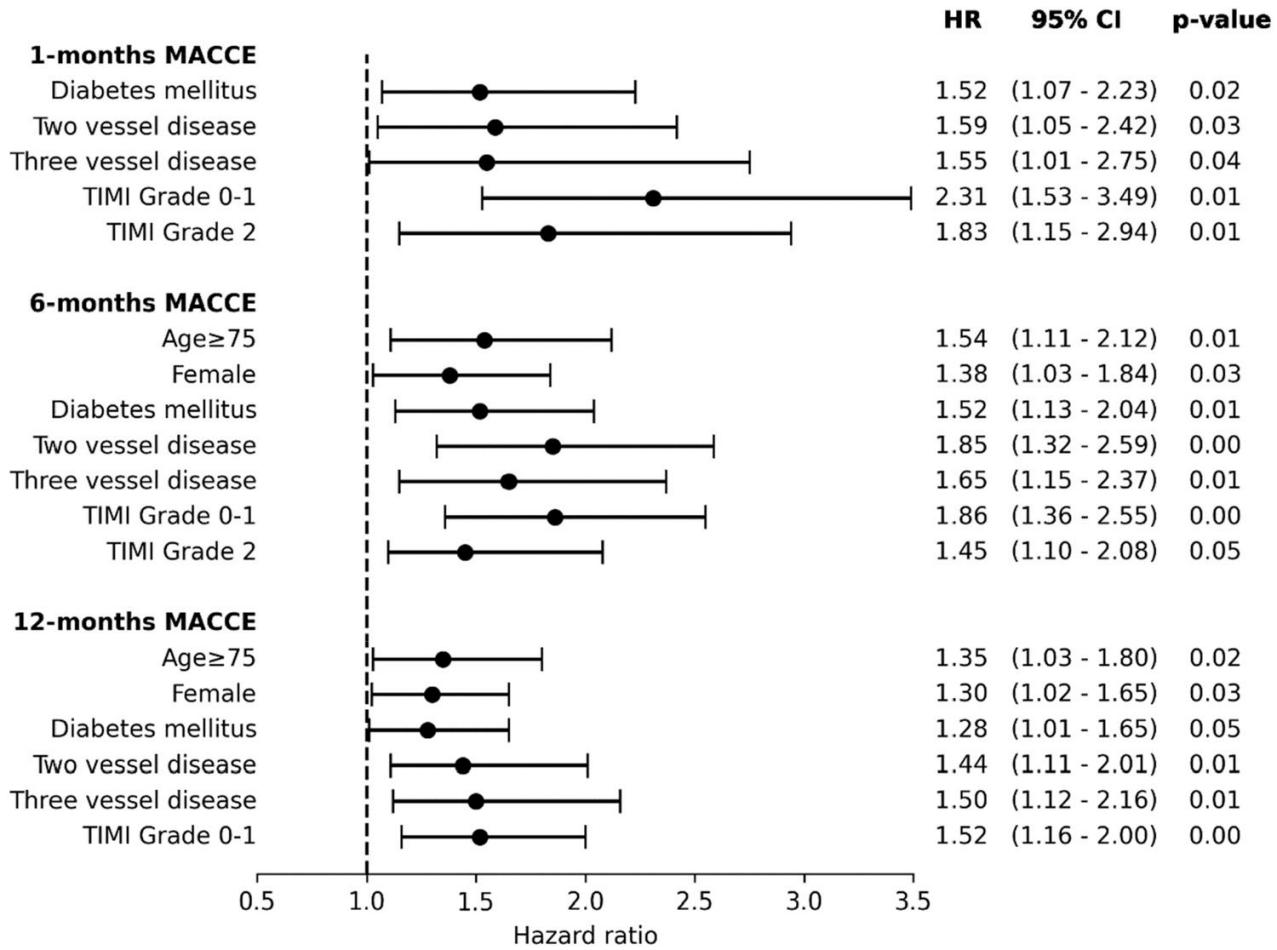
Multivariable analyses of associations between baseline variables and the occurrence of MACCE at 1, 6, and 12‐months after PCI. Only variables with significant associations with the occurrence of MACCE were included in the model. Abbreviations: 95% CI, 95% confidence interval; HR, hazard ratio; Ref, reference.

Multivariate analysis was conducted to identify the risk factors of MACCE occurrence. DM (HR 1.52, 95% CI 1.07–2.23), 2VD (HR 1.59, 95% CI 1.05–2.42), 3VD (HR 1.55, 95% CI 1.01–2.75), TIMI grade 0‐І (HR 2.31, 95% CI 1.53–3.49), TIMI grade П (HR 1.83, 95% CI 1.15–2.94) was identified as the independent predictor for 1‐months MACCE. Similar findings were observed for 6‐ and 12‐months MACCE. In addition, age and sex were also related to the increased occurrence of MACCE. However, the HR decreased over longer follow‐up periods, indicating a diminishing relative risk over time (Figure 1).

**Figure 2:**
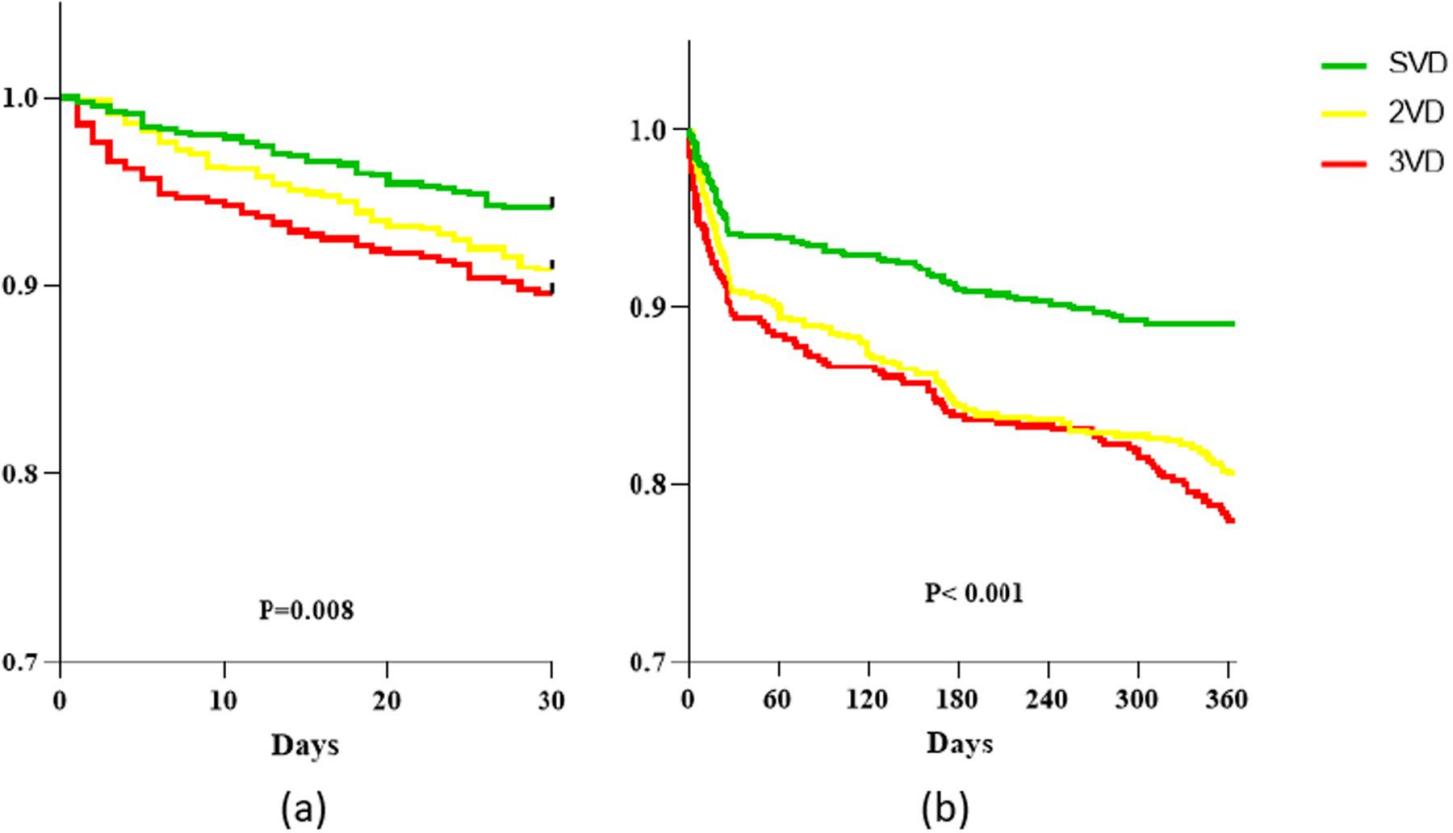
Kaplan–Meier curves evaluating the occurrence of MACCE based on the number of vessels involved. Kaplan–Meier curves (a) and (b) depict survival rates at 1 and 12‐month follow‐ups, respectively.

Kaplan–Meier survival curve analysis demonstrated a significant difference in MACCE‐free survival within 1, and 12‐months (log‐rank test, *p*< 0.001). MACCE occurrence was more frequent in patients with 2 or 3VD than in patients with SVD (Figure 2).

## 4 Discussion

In this cohort study, we aimed to determine the independent predictors of the development of MACCE in patients who underwent PCI in the East of Iran. We identified several predictors of MACCE, including age, female sex, DM, MVD, and TIMI flow post‐procedure. Our results indicated that the MACCE occurrence was 8.5% at 1 month, 13.7% at 6 months, and 17.9% at 12 months after PCI, which aligns with several large‐scale studies on after PCI outcomes. For instance, Ikebe et al. [11] and Keeley et al. [12] reported the occurrence of MACCE within 1 month as 5.4% and 13%, respectively. In different studies in Iran, the occurrence of MACCE in long‐term follow‐up after PCI was reported as 9.6% [1] and 12.7% [13]. In another study, Yang et al. reported the incidence of MACCE within 12 months after PCI to be 17.1% [7]. Madhavan et al., in a meta‐analysis study, found that MACCE occurred in 17.9% of post‐PCI patients within 12 months [14]. Additionally, an important consideration is the heterogeneity in the definitions of MACCE employed by different studies, which vary between three, four, and five components (three, four, or five components) across studies, which influences data collection methods [15]. As Bosco et al. highlighted, inconsistent definitions of MACCE can introduce bias in the comparative analysis of study outcomes, and should therefore be carefully considered by researchers [16]. In our study, MACCE was defined as all‐cause mortality, MI, CVA, and TVR.

Researchers have focused on evaluating prognosis and survival outcomes after interventions such as PCI while investigating the influence of various factors on the incidence of cardiovascular events after PCI. In the subsequent sections, we will review these factors in detail.

### 4.1 Age and MACCE

In our findings, age ≥ 75 years was a significant risk factor for MACCE, particularly at 6 and 12 months of follow‐up. In one study, older age was a risk factor for MACCE, and renal, neurological, and access‐site complications were all more frequent in the elderly (≥ 85 years) patients [17]. In studies by Shi et al. [18] and Khan et al. [19], older age was identified as one of the causes of higher MACCE rates, similar to our study. Older adults typically present with more comorbidities, complex coronary lesions, and frailty, all of which contribute to higher procedural and post‐procedural complication rates, resulting in increased MACCE occurrence [20]. This underscores the importance of personalized therapeutic approaches in managing elderly patients after PCI, particularly considering their higher susceptibility to ischemic complications.

### 4.2 Sex and MACCE

Unlike age, the role of sex in the outcome of PCI is a topic of ongoing debate. Otawa et al. [21] and Aghajani et al. [22] were the only studies that found females to be a significant protective factor for MACCE. In contrast, we found that female sex was an independent predictor of a higher risk of 6‐ and 12‐month MACCE after PCI. Biological and pathophysiological differences specific to females have been suggested as potential reasons for the higher MACCE rates observed in females compared to males. Several previous studies have attributed the higher occurrence of MACCE in females largely to older age and a higher prevalence of comorbidities compared with males [2]. One potential factor is estrogen, a protective hormone for the cardiovascular system, which is significantly reduced in postmenopausal women. Pathological factors, such as comorbidities and worse hemodynamic status, also play a critical role in the observed outcomes [23]. Jakobsen et al. similarly demonstrated that females experienced more complications and worse hemodynamic status compared with males [24]. A meta‐analysis further showed that women more frequently presented with adverse cardiovascular risk profiles, including comorbidities, atypical chest pains, and worse hemodynamic status, all of which were associated with a higher incidence of MACCE after PCI [2]. It is noteworthy that females had worse clinical outcomes, emphasizing the need for physicians to pay more attention to female patients in clinical practice. Furthermore, recognizing less‐studied confounding factors such as frailty, malnutrition, and physical activity, and exploring their interactions, could help clarify the complex role of sex in PCI outcomes in future research [25].

### 4.3 DM and MACCE

Previous studies have shown that DM has been consistently associated with an increased risk of MACCE after PCI, especially in patients with chronic total occlusions [26]. We found that DM was associated with an increased risk of MACCE in short‐ and long‐term follow‐ups. Similar results were also reported by Meliga et al. [27]. Several mechanisms have been proposed to explain the effect of DM on adverse outcomes after PCI. For example, reduced myocardial tolerance to ischemia due to poor collateral circulation may contribute to worse outcomes [28]. The PROSPECT study showed that patients with DM tend to have coronary atherosclerotic lesions characterized by a large necrotic core, thin‐cap fibroatheroma, and high calcium content. These characteristic lesions favor plaque instability and degradation, and predict future MACCE independent of myocardial ischemia [29].

### 4.4 HTN and MACCE

HTN is a common risk factor for CAD and a leading cause of mortality in older people [30]. Although in our study, the frequency of history of HTN in the 12‐month follow‐up was related to the occurrence of MACCE after PCI, but this relationship was not confirmed in multivariate regression models. In this regard, Chen et al. showed that the frequency of HTN was higher in the MACCE group, but there was no statistically significant relationship [31]. In contrast, Schröder et al. found that HTN was the only predictor of mortality after PCI, but the population was older than 75 years [32]. In another study, Yan et al. demonstrate that HTN is the predictor of increased MACCE risk [33]. Notably, their adjusted model consisted of fewer variables than other studies, which may have affected their results. It appears that, despite the established HTN effect on CAD, this factor is not recognized as a predictor of MACCE after PCI. Further studies are warranted to clarify its role.

### 4.5 BMI and MACCE

Obesity is recognized as a significant risk factor for cardiovascular disease and mortality [34]. However, research presents conflicting evidence regarding the association between BMI and MACCE. In our study, BMI was not significantly associated with the occurrence of MACCE. In a large‐scale cohort study on the Iranian population, Nalini et al. showed that BMI was a poor predictor of mortality and argued that BMI cannot differentiate between fat mass, muscle, or bone density, and cannot reflect body fat distribution [35]. In this regard, Choi et al. showed that abdominal obesity was associated with a higher risk of MACCE compared to general obesity without abdominal fat [36]. This finding may be explained by the limitation of BMI in representing body fat distribution; alternative markers, such as wrist circumference and abdominal circumference, are considered stronger predictors in this context.

### 4.6 Angiography Findings and MACCE

The presence of MVD, particularly two‐vessel disease (2VD) and three‐vessel disease (3VD), was another important predictor of MACCE across all follow‐up periods. Huang et al. [37] and Biondi‐Zoccai et al. [38] both showed that the presence of MVD is a well‐established predictor of adverse outcomes following PCI, as these patients tend to have more extensive coronary artery involvement and a higher burden of atherosclerosis, which increases the likelihood of recurrent ischemic events. This finding highlights the importance of aggressive management and close monitoring of these high‐risk patients. Interestingly, our findings demonstrated that pre‐procedural TIMI flow grades 0‐I were associated with a significantly increased risk of MACCE compared to TIMI grade III flow. Poor TIMI flow, which reflects suboptimal myocardial perfusion, has been previously associated with worse clinical outcomes, as reported by Mrdovic et al. [6]. Several studies have shown that patients with a TIMI flow grade < 1 before mechanical reperfusion fare worse than patients with an initial TIMI flow grade > 1 [39]. Achieving optimal reperfusion is critical in improving both short‐ and long‐term outcomes in patients undergoing PCI, as evidenced by the lower rates of MACCE in patients with better TIMI flow. In addition, De Luca et al. demonstrated that the benefit of preprocedural TIMI 3 patency was more pronounced in high‐risk patients [40].

### 4.7 Strengths and Limitations

To our knowledge, this is the first large registry‐based study with a 1‐year follow‐up of post‐PCI patients to rigorously adjudicate predictors of MACCE in East of Iran. This study examined the factors affecting MACCE in three time periods, which is unique in its kind. Additionally, our study included a broader age range, enhancing generalizability compared to previous studies that focused solely on middle‐aged and elderly individuals.

Despite its strengths, our study has several limitations. First, we lacked detailed information on after PCI medical adherence, particularly to antiplatelet therapy, which is known to significantly influence MACCE rates. Second, genetic, socioeconomic, and environmental factors, which may have contributed to variability in outcomes, were not evaluated in this analysis. Third, we did not analyze several complications following PCI such as contrast‐induced nephropathy, vascular access injuries, and bleeding. Fourth, follow‐up relied on phone interviews, and asymptomatic myocardial infarctions, transient ischemic attacks (TIAs), and subclinical target vessel revascularizations (TVRs) may have been under‐reported if patients did not seek medical attention or were not hospitalized. In addition, as a retrospective, single‐center registry‐based cohort drawn from a tertiary referral hospital, this study is subject to selection bias, referral‐center effects, and possible incompleteness of registry data; patients treated at Razi Hospital may not be fully representative of the wider PCI population, which limits generalizability. Because excluded patients had incomplete records, a formal comparison of their baseline characteristics with those of included patients was not feasible, and potential attrition bias therefore cannot be excluded. The definition of MACCE included TVR; however, information on stent type (bare‐metal vs. drug‐eluting, and first‐ vs. newer‐generation DES) was not consistently recorded; because these carry different risks of target‐vessel revascularization, their omission is a limitation of the TVR component of MACCE. Furthermore, associations were estimated using conventional multivariable Cox regression; we did not apply propensity score adjustment, inverse probability weighting, or matching. As an observational, registry‐based study, residual and unmeasured confounding cannot be excluded, and the reported predictors should be interpreted as associations rather than causal determinants. Finally, renal function variables, serum creatinine, eGFR, and chronic kidney disease status were not recorded in the registry dataset during the study period and could not be entered into the models, despite being recognized predictors of post‐PCI outcomes. Lastly, the relatively low incidence of MACCE in our patients limited the power of this study. We recommend that in the future this study be repeated with a larger sample size and the value of different prediction models be compared.

## 5 Conclusion

Our study provides valuable insights into the incidence and predictors of MACCE following PCI in an Iranian population. Age, female sex, DM, MVD, and poor TIMI flow were identified as independent risk factors for MACCE at various follow‐up intervals. Furthermore, targeted interventions for high‐risk populations, particularly elderly individuals and those with MVD, are crucial to enhancing clinical outcomes.

## Author Contributions

**Nahid Azdaki:** conceptualization, methodology, writing – review and editing, project administration, supervision, data curation, resources. **Seyed Ali Moezi Bady:** writing – review and editing, investigation, conceptualization, methodology, resources. **Toba Kazemi:** writing – review and editing, formal analysis, supervision, data curation, methodology, validation, resources. **Marzieh Azimizadeh:** writing – review and editing, formal analysis, visualization, methodology, writing – original draft, software, conceptualization. **Hamidreza Mohammadi:** writing – review and editing, writing – original draft, visualization, methodology, software. **Alireza Khosravi Farsani:** writing – review and editing, validation, supervision, data curation. **Nizal Sarrafzadegan:** writing – review and editing, supervision, data curation, investigation, validation, resources. **Maryam Boshtam:** writing – review and editing, data curation, software. **Pedro Manuel Marques‐Vidal:** writing – review and editing, validation.

## Funding

The authors have nothing to report.

## Ethics Statement

The present study was approved by the Birjand University of Medical Sciences (ethics code: IR.BUMS.REC.1401.331) and conducted based on the Declaration of Helsinki on medical research.

## Consent

Informed consent was obtained from eligible individuals. The data collection process ensured complete anonymity, with no personally identifying information included in the individual data.

## Conflicts of Interest

The authors declare no conflicts of interest.

## Transparency Statement

The lead author, Marzieh Azimizadeh, affirms that this manuscript is an honest, accurate, and transparent account of the study being reported; that no important aspects of the study have been omitted; and that any discrepancies from the study as planned (and, if relevant, registered) have been explained.

## Data Availability

The data that support the findings of this study are available from the corresponding author upon reasonable request.
